# Association between physical activity, weight – adjusted waist index, and all – cause mortality in Chinese older adults: a national community – based cohort study

**DOI:** 10.5114/biolsport.2025.151659

**Published:** 2025-07-07

**Authors:** Kexin Ren, Yuan Tao, Meihong Wang

**Affiliations:** 1 College of Physical Education, Jilin Normal University, 1301, Hai Feng Street, Tiexi District, Siping City, Jilin Province, China; 2 College of Mathematics and Computer, Jilin Normal University,1301, Hai Feng Street, Tiexi District, Siping City, Jilin Province, China

**Keywords:** Physical activity, WWI, CLHLS, Older adults, All-cause mortality

## Abstract

Enhancing physical activity and managing body weight are crucial for addressing aging-related challenges. However, research on the relationship between physical activity, Weight-Adjusted Waist Index (WWI), and all-cause mortality is limited. This study aims to explore these interactions and their impact on elderly health. Data from the Chinese Longitudinal Healthy Longevity Survey (CLHLS) for 2011–2018 included 7,034 residents aged ≥ 60 years. We utilized Cox proportional hazard models to assess the relationships between physical activity, WWI, and all-cause mortality, supplemented by subgroup analyses and interaction tests. We did a mediation analysis to assess how much of the effect of physical activity on survival status was mediated through WWI. Active individuals and those transitioning from inactive to active lifestyles exhibited significantly lower all-cause mortality risks, with reductions of 26% (HR = 0.74, CI: 0.65–0.83) and 9% (HR = 0.91, CI: 0.83–0.99), respectively. A positive correlation was found between WWI and all-cause mortality, with a threshold of 11.38 cm/√kg indicating increased risk. Although no interaction between physical activity and WWI was observed (P = 0.462), mediation analysis showed that 3.06% of the effect of physical activity on survival status was mediated through WWI. Maintaining physical activity or transitioning from a sedentary lifestyle to an active one can significantly reduce all-cause mortality in the elderly. Moreover, high WWI is associated with an increased risk of death. Importantly, WWI partially mediates the relationship between physical activity and death, shedding light on why physical activity reduces mortality and reinforcing the need for health promotion strategies tailored to the elderly population.

## INTRODUCTION

With the burgeoning aging population in China, the nation is grappling with the multifaceted challenges of an aging society. According to the National Bureau of Statistics of China, the proportion of individuals aged 60 and above is projected to reach over 35% by 2050 [1]. This demographic shift brings to the forefront the importance of understanding factors that contribute to healthy aging and the reduction of age-related morbidity and mortality.

Physical activity is widely recognized for its salutary effects on health, particularly in older adults. Numerous epidemiological studies have underscored the role of regular exercise in mitigating the risk of all-cause mortality [2, 3, 4].This includes a reduction in premature death from all causes [5], a decreased risk of 26 different types of cancer [6], and a lower risk of heart disease, which is one of the leading causes of death globally [7]. However, there are few natiomnal community-based cohort studies that address the effects of physical activity on all-cause mortality in Chinese older adults. A study by Yin R et al. based on CLHLS data demonstrated that maintaining regular physical activity or shifting from inactivity to activity was consistently associated with longer survival in the elderly population, but for a study population aged 80 years and older [8].

Concurrently, there is a growing interest in the relationship between adiposity measures and health outcomes in the elderly. The Weight-Adjusted Waist Index (WWI), a novel anthropometric indicator, has emerged as a promising tool for assessing obesity-related risks [9]. The index, calculated by dividing waist circumference by the square root of weight, is posited to better reflect the distribution of abdominal fat, which is more closely associated with adverse health outcomes [10]. Recent studies, particularly in Chinese populations, have begun to unravel the association between WWI and health outcomes, such as hypertension incidence [11] and cardiovascular mortality [12]. Additionally, another study reported a nonlinear relationship between WWI and all-cause mortality, suggesting that both very high and very low WWI values may confer increased mortality risk [13].

Despite the individual associations of physical activity and WWI with health outcomes, there is a dearth of research examining their combined effects on all-cause mortality in the Chinese older adult population. Existing literature has yet to explore the interplay between these factors within a national, community-based cohort study framework. Such research is vital, as it can provide insights into the complex interactions between lifestyle factors and health in the context of a rapidly aging China.

This study, based on the Chinese Longitudinal Healthy Longevity Survey (CLHLS), aimed to investigate the independent and combined effects of physical activity and WWI on mortality risk.The findings of this research have the potential to inform public health strategies and clinical guidelines aimed at promoting healthy aging and reducing premature mortality among China’s elderly population.

## MATERIALS AND METHODS

### Data sources

The data for this study come from the the Chinese Longitudinal Healthy Longevity Survey (CLHLS) [14], the largest cohort study of the elderly population in China, organised by the Centre for Healthy Ageing and Development at Peking University and the National Institute for Development Research. The CLHLS covered 23 provinces, municipalities, and autonomous regions, with a cumulative total of 113,000 household interviews, and randomly selected about half of the cities and counties as research sites in the 22 research provinces (excluding Hainan Province). The survey was approved by the Institutional Review Board of Peking University(IRB00001052-13074). All participants or their legal representatives provided written informed consent.

**FIG. 1:**
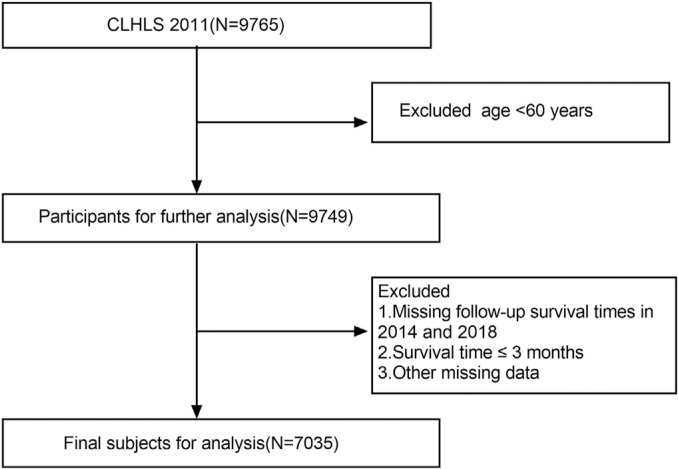
Flow chart of this analysis based on CLHLS 2011-2018.

We employed a sample comprising individuals aged 60 and above from the 2011 follow-up study, and all participants underwent subsequent evaluations in 2014 and 2018, with continuous monitoring extending until their demise, loss of follow-up, or the culmination of the study. Participants lacking complete records or surviving ≤ 3 months were excluded from the analysis.The final sample size for analysis is 7,035 (see Figure 1).

### Variable Measurement

#### Exposure

In the survey, participants were asked “Do you exercise frequently at present?” and “Did you exercise frequently in the past?” Based on their responses to these two questions, participants were categorised into four groups: 1) physically active if they exercised frequently both in the past and now, 2) physically inactive if they did not exercise frequently either in the past or present, 3) inactive-to-active if they did not exercise frequently in the past but do so now, and 4) activeto- inactive if they exercised frequently in the past but do not exercise currently [15].

WWI is obtained by dividing the waist circumference (cm) by the square root of the body weight (kg) [9]. Physical examinations were performed during face-to-face interviews. Trained staff measured baseline weight and waist circumference following a standardized protocol. Participants were weighed in light clothing, with measurements taken to the nearest 0.1 kg. Waist circumference was measured using a flexible tape at the midpoint between the lowest rib and the iliac crest, rounded to the nearest 1 cm.

### Outcome

The study’s outcome was all-cause mortality. In the second and third surveys, data were gathered regarding the participants’ survival status and date of death. If the exact day was unavailable, the 15^th^ of the month was used as the assumed date of death. Participants who were still alive or lost to follow-up were censored at their last point of contact.

### Covariates

Based on previous studies of physical activity and mortality, as well as WWI and mortality, we included a variety of covariates that might influence the results: age, gender, birth place, educational background, marital status, smoking [16], drinking [17], sleep quality [18], waist circumference [19], Self-assessment of health [20], medical insurance [21], economic state [22].

It is important to note that the economic status of participants in the CLHLS cohort was self-reported and evaluated using a Likertstyle questionnaire. Specifically, respondents were asked: “How would you rate your economic status relative to other local residents?” Response categories included:Very rich, Rich, So-so, Poor, Very poor. For the purpose of data analysis, we combined responses Very rich and Rich into a single category labeled “Affluent and above,” which was assigned a value of “1.” Responses So-so, Poor, and Very poor were merged into another category labeled “Average and below,” which was assigned a value of “0.”

### Statistical analysis

Participant demographics were analyzed across subgroups of physical activity using chi-square tests and ANOVA. We employed Cox proportional hazards models to examine the relationship between physical activity, WWI, and all-cause mortality.The result was expressed as hazard ratios (HR) and 95% confidence intervals (CI).To account for potential confounders, we established three models: Model 1 was unadjusted; Model 2 was adjusted for age, sex, residence, educational background, and marital status; and Model 3 was further adjusted for age, sex, educational background, marital status, alcohol consumption, smoking status, Self-assessment of health, medical insurance, and socioeconomic status.The restricted cubic spline (RCS) curves were employed to analyse the non-linear relationship between physical activity and all-cause mortality, and similarly between WWI and all-cause mortality; and finally, mediating effect analysis was conducted to explore the mechanism of WWI’s role in the relationship between physical activity and survival status. All statistical analyses were performed using Stata(version 17), R (version 4.3.2) or Zstats (https://www.medsta.cn/).

## RESULTS

### Baseline characteristics

The socio-demographic characteristics, lifestyle habits, and socioeconomic factors of the participants categorized by changes in physical activity are presented in Table 1. Among the 7,034 participants, nearly half (N = 3,433, 48.8%) remained inactive, while fewer than 20% (N = 1,149, 16.3%) had always been active. Additionally, 22.8% (N = 1,603) transitioned from inactive-to- active, and 12.1% (N = 849) shifted from active-to-inactive. The mean age was significantly lower in both the active group and the inactiveto-active group compared to the other two groups (P < 0.001). Females dominated the inactive group (58.52%), while males were more prevalent in the active group (59.18%). Participants who either maintained or adopted exercise had higher self-assessed health scores compared to the two groups that remained inactive (P < 0.001). Furthermore, we categorized the WWI of the different exercise groups into quartiles, revealing a significant difference in the values among the groups (P < 0.001).

**TABLE 1:** Baseline Characteristics and Changes in Physical Activity Status Among Older Adults in the CLHLS 2011–2018

Variables	Total (n = 7034)	inactive (n = 3433)	active (n = 1149)	Inactive-to-active (n = 1603)	Active-to-inactive (n = 849)	*P*
**Age, Mean ± SD**	84.35 ± 10.88	85.14 ± 11.38	82.13 ± 10.08	82.66 ± 9.99	87.30 ± 10.40	**< .001**

**Gender, n(%)**						**< .001**
Male	3268 (46.46)	1424 (41.48)	680 (59.18)	756 (47.16)	408 (48.06)
Female	3766 (53.54)	2009 (58.52)	469 (40.82)	847 (52.84)	441 (51.94)

**Ethnic, n(%)**						**0.008**
Non-Han	419 (5.96)	228 (6.64)	48 (4.18)	102 (6.36)	41 (4.83)
Han	6615 (94.04)	3205 (93.36)	1101 (95.82)	1501 (93.64)	808 (95.17)

**Birth Place, n(%)**						**< .001**
Rural	6281 (89.29)	3227 (94.00)	871 (75.81)	1458 (90.95)	725 (85.39)
Urban	753 (10.71)	206 (6.00)	278 (24.19)	145 (9.05)	124 (14.61)

**Marital, n(%)^a^**						**< .001**
Others	4273 (60.75)	2180 (63.50)	592 (51.52)	941 (58.70)	560 (65.96)
Married	2761 (39.25)	1253 (36.50)	557 (48.48)	662 (41.30)	289 (34.04)

**Education, n(%)**						**< .001**
lliterate	3896 (55.39)	2222 (64.72)	371 (32.29)	858 (53.52)	445 (52.41)
Primary	2270 (32.27)	968 (28.20)	444 (38.64)	565 (35.25)	293 (34.51)
Secondary	739 (10.51)	219 (6.38)	268 (23.32)	163 (10.17)	89 (10.48)
University and above	129 (1.83)	24 (0.70)	66 (5.74)	17 (1.06)	22 (2.59)

**Drink, n(%)**						**0.034**
No	5711 (81.19)	2819 (82.11)	928 (80.77)	1264 (78.85)	700 (82.45)
Yes	1323 (18.81)	614 (17.89)	221 (19.23)	339 (21.15)	149 (17.55)

**Econnomic State, n(%)**						**< .001**
Average and below	5780 (82.17)	2949 (85.90)	852 (74.15)	1273 (79.41)	706 (83.16)
Affluent and above	1254 (17.83)	484 (14.10)	297 (25.85)	330 (20.59)	143 (16.84)

**Medical Insurance, n(%)**						**< .001**
No	1191 (16.93)	459 (13.37)	296 (25.76)	243 (15.16)	193 (22.73)
Yes	5843 (83.07)	2974 (86.63)	853 (74.24)	1360 (84.84)	656 (77.27)

**WWI, n(%)^b^**						**< .001**
Q1	1768 (25.14)	880 (25.63)	282 (24.54)	404 (25.20)	202 (23.79)
Q2	1765 (25.09)	853 (24.85)	321 (27.94)	388 (24.20)	203 (23.91)
Q3	1749 (24.86)	791 (23.04)	337 (29.33)	410 (25.58)	211 (24.85)
Q4	1752 (24.91)	909 (26.48)	209 (18.19)	401 (25.02)	233 (27.44)

**Waist, mean ± SD**	81.72 ± 18.83	80.43 ± 18.73	84.21 ± 13.63	82.23 ± 12.33	82.61 ± 31.09	**< .001**

**Sleep quality, Mean ± SD**	2.32 ± 0.96	2.37 ± 0.94	2.16 ± 0.94	2.30 ± 0.98	2.36 ± 0.98	**< .001**

**Health, Mean ± SD**	2.40 ± 0.92	2.31 ± 0.90	2.63 ± 0.91	2.48 ± 0.91	2.30 ± 0.95	**< .001**

Mean ± SD for continuous variables: the P value was calculated by ANOVA; (%) for categorical variables: the P value was calculated by Chi-square test;

a Others include widowed, separated, divorced, or never married;

b Q is for Quartile.

### Association between Physical Activity, WWI and All-cause Mortality

We conducted a comprehensive analysis utilizing Cox proportional hazards regression to explore the relationship between diverse physical activity patterns and all-cause mortality, as detailed in Table 2. Both the unadjusted and covariate-adjusted results robustly demonstrated a statistically significant decrease in the hazard ratio (HR) among individuals classified as active, as well as those transitioning from inactive-to-active statuses (p < 0.001). Specifically, in Model 3, our findings revealed that the HR for the active group stood at 0.74 (95% CI: 0.65–0.83), translating to a remarkable 26% reduction in the risk of mortality compared to their less active counterparts. Furthermore, for those who transitioned from an inactive to an active lifestyle, the HR was 0.91 (95% CI: 0.83–0.99), signifying a potential 9% reduction in the risk of death, emphasizing the positive impact of altering exercise habits.

**TABLE 2:** Association between physical activity, WWI, and All-Cause Mortality in older adults: Results from multivariable-adjusted Cox models in CLHLS 2011–2018.

Variables	Model 1		Model 2		Model 3	

	HR (95%CI)	*P*	HR (95%CI)	*P*	HR (95%CI)	*P*
**Physical Activity**						
Inactive	1.00 (Reference)		1.00 (Reference)		1.00 (Reference)
Active	0.63 (0.56 ~ 0.71)	**< .001**	0.72 (0.64 ~ 0.81)	**< .001**	0.74 (0.65 ~ 0.83)	**< .001**
Inactive-to-active	0.82 (0.75 ~ 0.90)	**< .001**	0.90 (0.82 ~ 0.99)	**0.025**	0.91 (0.83 ~ 0.99)	**0.049**
Active-to-inactive	1.21 (1.09 ~ 1.34)	**< .001**	1.10 (0.99 ~ 1.22)	0.085	1.11 (1.00 ~ 1.24)	0.053

**WWIa**						
Q1 (< 10.58 cm/√kg)	1.00 (Reference)		1.00 (Reference)		1.00 (Reference)
Q2 (10.58 ~ 11.38 cm/√kg)	0.99 (0.89 ~ 1.09)	0.775	1.00 (0.90 ~ 1. 10)	0.924	1.13 (1.01 ~ 1.26)	**< .001**
Q3 (11.38 ~ 12.30 cm/√kg)	1.00 (0.90 ~ 1. 11)	0.965	0.94 (0.85 ~ 1.04)	0.235	1.15 (1.02 ~ 1.29)	**0.033**
Q4 (≥ 12.30 cm/√kg)	1.24 (1. 12 ~ 1.37)	**< .001**	1.04 (0.94 ~ 1. 15)	0.444	1.43 (1.25 ~ 1.63)	**0.023**

HR: Hazard Ratio, CI: Confidence Interval, aQ is for Quartile. Model 1: Crude; Model 2: Adjust: age, gender, ethnic, birth place, education, marital; Model 3: Adjust: age, gender, ethnic, birth place, education, marital, econnomic state, medical insurance, waist, sleep quality, health.

Moreover, we conducted a thorough examination of the correlation between WWI and overall mortality, with the outcomes presented in Table 2. Prior to adjusting for confounding variables, a pronounced elevation in mortality risk was observed exclusively in the fourth quartile (Q4, the highest category) compared to the first quartile (Q1, the lowest category), reaching statistical significance (p < 0.001), with a hazard ratio (HR) of 1.24 (95% CI: 1.12 to 1.37). Subsequent to comprehensive adjustment for covariates, notable increases in mortality risk emerged for both the second (Q2) and third (Q3) quartiles (P < 0.05), while the risk in the fourth quartile (Q4) demonstrated an exceedingly significant surge (P < 0.001). In summary, a graded increase in mortality risk was observed with progressive elevation in WWI quartiles, with respective increments of 13% (Q2), 15% (Q3), and a substantial 43% (Q4), underscoring the heightened risk associated with higher WWI levels.

**FIG. 2:**
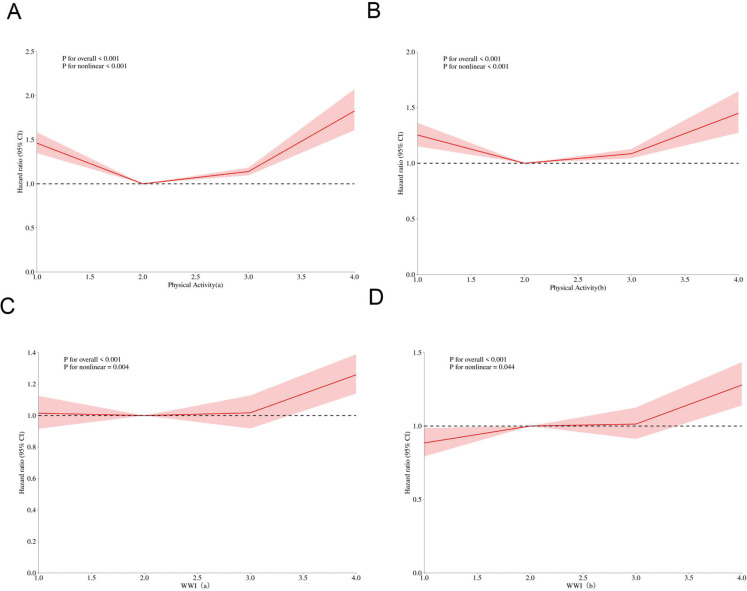
Nonlinear associations of physical activity and WWI with all-cause mortality among Chinese older adults. Note: The solid red line represents the smooth curve fit between variables. Red bands represent the 95% of confidence interval from the fit. (A) and (B) represent the relationship between physical activity and all-cause mortality. (A) does not adjust for covariates, while (B) does. (C) and (D) represent the relationship between WWI and all-cause mortality.(C) does not adjust for covariates, while (D) does.

In order to delve nonlinear associations among physical activity, WWI, and all-cause mortality, we crafted RCS curves(Fig. 2). Regarding the relationship between physical activity and all-cause mortality, our analysis revealed a pronounced statistical correlation (P < 0.001 for the overall analysis), both prior to and subsequent to adjusting for covariates. Notably, the P-values for non-linearity in (A) and (B) of Fig. 2, which depict this relationship, remained below 0.001, conclusively demonstrating a nonlinear association between physical activity levels and all-cause mortality. Elderly individuals who maintain an active lifestyle consistently exhibit the lowest all-cause mortality rates. Moreover, those transitioning from inactivity to an active lifestyle display a mortality rate slightly elevated compared to the consistently active group but significantly lower than those who remain inactive or move from active-to-inactive.

Similarly, the link between WWI (Waist-to-Weight Index) and allcause mortality manifested a statistically significant correlation (P < 0.001) before and after covariate adjustment. However, upon covariate adjustment, the strength of evidence for nonlinearity weakened, as evidenced by a notable increase in the P-value for non-linearity (from P < 0.001 to P < 0.044), suggesting that while the nonlinear relationship persists, its statistical significance diminishes. (C) and (D) of Fig. 2 highlight a distinct inflection point corresponding to Q3 of WWI, revealing that when WWI surpasses 11.38 cm/√kg, there is a marked increase in all-cause mortality rates.

### Subgroup Analyses for Association Between Physical Activity and All-Cause Mortality

**FIG. 3:**
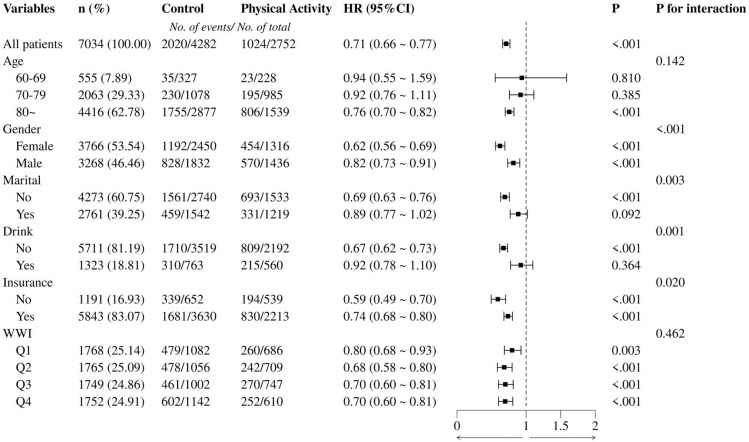
Subgroup analysis of the associations between physical activity and all-cause mortality risk among older adults in China (CLHLS 2011-2018).

To assess whether the association between physical activity and all-cause mortality was consistent across populations, subgroup analyzes and interaction tests stratified by age, gender, marial, drink, medical insurance and WWI were performed. The physical activity group shown in Figure 3 was derived from the active and inactive-to- active groups, and the control group was derived from the inactive and active-to- inactive groups. Subgroup analyses revealed significant interactions between physical activity and gender, marital status, drinking habits, and insurance status. Notably, females (HR = 0.62) and non-drinkers (HR = 0.67) exhibited a more pronounced reduction in risk. Age and WWI quartiles did not show significant interactions with physical activity, although a consistent reduction in risk was observed across all age groups and WWI categories.

### Mediating effects of WWI between physical activity and survival states

**FIG. 4:**
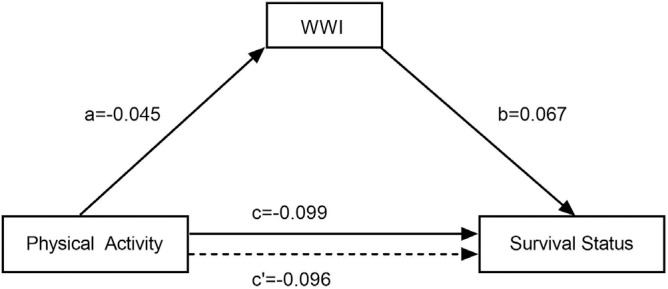
Mediation model of WWI in the relationship between physical activity and survival status among older adults in China (CLHLS 2011-2018) Note: a: effect value of physical activity on WWI, b: effect value of WWI on survival status, c: total effect value of physical activity on survival status, c’: direct effect value of physical activity on survival status. The values (a, b, c, c’) represent the regression coefficients from the mediation analysis.

Following stepwise regression, linear regression analyses were conducted with physical activity as the independent variable, survival status as the dependent variable, and WWI as the mediating variable. The results showed that physical activity had an effect on both WWI and survival status (i.e., c: β = -0.099, P < 0.001; a: β = -0.045, P < 0.001), and after the introduction of WWI, physical activity, WWI had an effect on survival status (i.e., c’: β = -0.096, P < 0.001; b: β = 0.067, P < 0.001). After establishing the mediation model, it showed that the total effect value of physical activity on survival status was -0.099, and the direct effect value was -0.096, and ab was the same sign as c’, which suggests that there is a partially mediated effect of WWI between physical activity and survival status, as shown in Figure 4.

To robustly validate the mediating effects of WWI in the relationship between physical activity and survival status among older adults, we conducted hypothesis testing employing the nonparametric percentile Bootstrap method. Setting Bootstrap random sampling 5,000 times, the results show that none of the Bootstrap confidence intervals contain 0, indicating that the partial mediating effect of WWI on the impact of physical activity on the survival status of elderly people has statistical significance, although the proportion of mediating effect to the total effect is only 3.06%, see Table 3.

**TABLE 3:** Bootstrap Analysis of the Mediation Effect of WWI on the Relationship Between Physical Activity and Survival Status in Older Adults: CLHLS 2011–2018.

Effect	Coefficient	Std. Err.	95% CI	P
Indirect Effect	-0.003	0.001	-0.001 ~ -0.005	< 0.001
Direct Effect	-0.096	0.011	-0.073 ~ -0. 120	< 0.001
Total Effect	-0.099	0 .012	-0.076 ~ -0. 123	< 0.001
PM,%	3.06

PM, percent mediation

## DISCUSSION

Based on our analysis of CLHLS data, it is evident that physical activity plays a crucial role in enhancing longevity among older adults, and the transition from inactivity to activity is equally beneficial. In exploring the nexus between physical activity and all-cause mortality, particularly in the elderly, many studies have provided consistent insights as presented in this article. For example, a longitudinal study of 85,545 elderly Australians found that a high-quality diet combined with high levels of moderate-to-vigorous physical activity (MVPA) significantly reduced risks of cardiovascular disease (CVD) and allcause mortality [23]. The National Health and Nutrition Examination Survey (NHANES) 2011–2014 data emphasized that objective measures of physical activity, above age, are the strongest predictors of all-cause mortality [24].A retrospective cohort study utilizing Korean National Health and Nutrition Examination Survey (KNHANES)-mortality linked data underscored the joint influence of physical activity and socioeconomic status (SES) on mortality, revealing a notable decrease in mortality risk among older adults with low SES who engage in regular physical activity [25]. Similarly, among low-income older Americans, a cohort study showed that high sitting time is an independent risk factor for all-cause and CVD mortality, while leisure-time physical activity (LTPA) mitigated this risk [26].In the Indian context, the Longitudinal Aging Study evidence suggests that adequate physical activity significantly lowers the risk of CVD among the elderly, emphasizing the importance of regular exercise in reducing CVD burden [27].Collectively, these findings highlight the pivotal role of physical activity in promoting longevity and reducing all-cause mortality in the elderly, transcending geographical and socioeconomic barriers. The shift from a sedentary to an active lifestyle not only extends life expectancy but also has significant public health implications. Therefore, we recommend that public health policies should prioritize the promotion of physical activity among older adults through targeted interventions such as community-based exercise programs, accessible fitness facilities, and public awareness campaigns.

WWI has garnered attention as an emerging indicator for assessing obesity. Experimental findings indicate that a reduction in WWI significantly decreases the risk of multimorbidity in older adults [28] and is positively associated with all-cause mortality in this population [13].All these results strongly support the premise of this paper that higher WWI values are associated with an increased risk of death. Furthermore, we identified that a WWI threshold of ≥ 11.38 cm/√kg may indicate an elevated risk of death among Chinese older adults, as demonstrated by the RCS curve. This finding slightly differs from those of other studies.For example, according to the NHANES database, a study of a non-Asian population aged 18 to 80 years found that when WWI exceeded 10.46 cm/√kg, all-cause mortality increased by 20% for each unit increase in WWI [HR = 1.20, 95% CI: (1.08, 1.33)] [29]. Additionally, a cohort study conducted in rural China recruited 10,338 non-hypertensive participants aged 18 years and older in Henan Province. This study found a positive association between WWI and the prevalence of hypertension over a subsequent six-year follow-up, revealing a significantly higher odds ratio (OR) for developing hypertension, particularly when WWI was ≥ 10.91 cm/√kg [OR = 1.50, 95% CI: 1.24–1.82] [29]. The subtle differences in WWI thresholds may be attributed to the fact that the study population in this paper comprised individuals aged 60 years and older.

Given that both physical activity as well as WWI have been shown to be strongly associated with all-cause mortality, a thought-provoking question is: Is there some potential correlation between the two? In order to explore this topic, subgroup analyses and interaction analyses were carefully designed with the aim of revealing possible subtle links between them.The results of the study showed significant interaction effects between physical activity and a range of socio-demographic factors and lifestyle habits, including gender, marital status, alcohol consumption, and health insurance status, when allcause mortality was taken into account. These findings not only enrich our knowledge of health risk factors, but also highlight the importance of individual differences in influencing health outcomes.However, it is worth noting that when exploring whether there was an interaction between physical activity and WWI, our analyses did not find a statistically significant association between the two (P for interacton = 0.462).It is important to note that the assessment of physical activity in our study was based on self-reported data, which could introduce potential bias [30].

Further, we analysed in depth the mediating effect of WWI between physical activity level and survival status.By constructing a mediating effect model, we found that WWI partially mediated this relationship. Although the proportion of the mediating effect was relatively low, at 3.06%, this finding is still clinically significant. Physical activity may have an indirect positive impact on all-cause mortality by reducing WWI values, which in turn may contribute to lowering all-cause mortality.Furthermore, despite the low proportion of the mediating effect, it serves as a reminder that other potential mediating variables or pathways should not be overlooked when exploring the relationship between physical activity and health outcomes. Future research could further explore how physical activity affects all-cause mortality through other mechanisms, such as improving cardiovascular health [31], reducing the inflammatory response [32], strengthening the immune system [33], promoting mental health [34], and improving metabolic function [35], and how these mechanisms interact with WWI.

Our study adds a novel perspective to the existing literature by specifically examining the role of Weight-Adjusted Waist Index (WWI) in mediating the relationship between physical activity and all-cause mortality in a Chinese elderly population. This focus on WWI, as opposed to more commonly used indicators like BMI, provides a unique contribution to understanding obesity-related health risks in this demographic.

## Limitation

This study acknowledges several limitations. Firstly, the reliance on self-reported physical activity data introduces potential biases, such as recall and social desirability, which may compromise the accuracy of the reported levels. To mitigate this, future research should consider integrating more objective measures, like wearable devices, to enhance data reliability. Additionally, the lack of comparison with other anthropometric indicators, such as BMI, limits the comprehensiveness of our findings. Including these indicators in future studies would facilitate a more robust assessment of the predictive power of Weight-Adjusted Waist Index (WWI) relative to other obesity indicators. Lastly, regarding the mediating role of WWI, we must consider the possibility of reverse causality. For example, poor health could lead to both reduced physical activity and increased central adiposity. To better understand these complex relationships, it is recommended that future studies employ longitudinal designs with multiple time points.

## CONCLUSIONS

In conclusion, our study of older adults in China shows that maintaining physical activity or shifting from a sedentary lifestyle to an active one can reduce all-cause mortality. Conversely, a higher Weight-Adjusted Waist Index (WWI) is linked to an increased risk of all-cause mortality.The WWI seems to have a partial mediating effect between physical activity and death, shedding light on why physical activity reduces the risk of death. Further research is necessary to investigate additional mechanisms through which physical activity may lower the risk of death. The findings provide scientific evidence for developing health promotion strategies aimed at the elderly population, highlighting the importance of regular physical activity and maintaining a healthy weight to prevent premature death.

## Data Availability

Raw data for this article is available from the CLHLS website (https://opendata.pku.edu.cn). The datasets used and/or analyzed during the current study are available from the first author on reasonable request (First author: Kexin REN, E-mail: jlsdrkx@163.com).
